# Orthostatic hypotension in continuous-flow LVADs: Baroreflex unloading, preload dependence, and a phenotype-specific framework

**DOI:** 10.1016/j.jhlto.2026.100710

**Published:** 2026-10-16

**Authors:** Alexandria Danyluk, Ivan Nenadic, Stephen P. Wright, Jason Guichard, Jonathan Howlett, Satish R. Raj, Mark N. Belkin, Andrew J. Sauer, Hirak Shah, Susanna Mak, Marat Fudim

**Affiliations:** a Duke Heart Center, Duke University Hospital, Durham, NC; b Centre for Heart Lung Innovation, Providence Research, The University of British Columbia and St. Paul’s Hospital, Vancouver, BC, Canada; c Department of Medicine, Division of Cardiology, Section for Advanced Heart Failure, Pulmonary Hypertension, and Mechanical Circulatory Support, Prisma Health-Upstate, Greenville, SC; d Department of Physical Therapy, Faculty of Medicine, The University of British Columbia, Vancouver, BC, Canada; e Libin Cardiovascular Institute, University of Calgary and Alberta Health Services, Calgary, AB, Canada; f Section of Cardiology, University of Chicago, Chicago, IL; g Saint Luke’s Mid America Heart Institute, Kansas City, MO; h Department of Cardiovascular Medicine, University of Kansas Medical Center, Kansas City, KS; i Division of Cardiology, Department of Medicine, University of Toronto, Toronto, ON, Canada

**Keywords:** orthostatic hypotension, left ventricular assist device (LVAD), continuous-flow LVAD (CF-LVAD), mechanical circulatory support (MCS), advanced heart failure

## Abstract

Orthostatic hypotension (OH) is an underrecognized consequence of continuous-flow left ventricular assist device (CF-LVAD) therapy, affecting 12–30% of patients. Reduced arterial pulsatility during CF support creates a distinct autonomic environment characterized by impaired baroreceptor stimulation, defective sympathetic vasoconstriction, exaggerated venous pooling, and extreme preload dependence. Traditional blood pressure assessments often fail to identify OH in this population, and resting hemodynamics rarely reveal the underlying mechanism.

In this perspective piece, we propose a two-phenotype, physiology-based framework for OH specific to CF-LVAD patients. We synthesize current knowledge of baroreflex biology, vascular capacitance, and pump dynamics to describe how reduced pulsatility disrupts cardiovascular responses to upright posture. Within this framework, we define two dominant mechanistic phenotypes: (1) autonomic dysfunction-predominant failure, where impaired reflexes drive orthostatic venous pooling and LV underfilling, and (2) right ventricular (RV) failure–limited forward flow, where RV dysfunction constrains preload delivery to the pump despite preserved autonomic tone.

We outline how integrating autonomic data with LVAD hemodynamics and posture-linked imaging—specifically dynamic changes in venous capacitance, inferior vena cava behavior, left ventricular end-diastolic dimension, and pump performance—forms a coherent diagnostic and management pathway. This physiology-first approach allows clinicians to distinguish between preload failure and RV-limited flow at the bedside and tailor therapy accordingly.

A mechanistic understanding of OH in CF-LVAD patients is essential for accurate diagnosis, preventing suction events, optimizing device settings, and reducing morbidity through targeted, mechanism-specific management.

## Introduction

Orthostatic hypotension (OH)^1^ is a common but often underappreciated complication of continuous-flow left ventricular assist device (CF-LVAD) therapy, with reported prevalence ranging from 12%, based on symptoms, to nearly 30% when formal orthostatic testing is performed.^2^ Although OH is well-described in the general heart failure population, most prior work in CF-LVAD patients consists of isolated case reports, small case series, or extrapolation from non-LVAD OH paradigms. What is lacking is a unifying, physiology-based framework tailored to the unique hemodynamic and autonomic environment created by continuous-flow support, as well as a practical diagnostic and management algorithm for bedside use. Importantly, orthostatic symptoms in CF-LVAD patients often reflect posture-dependent preload delivery and baroreflex unloading rather than conventional “blood pressure variability,” and bedside posture-linked imaging combined with device parameters can distinguish opposing mechanisms that otherwise appear clinically indistinguishable.

The mechanisms of OH during CF-LVAD support differ fundamentally because of alterations in arterial pulsatility, baroreflex function, venous capacitance, and the intrinsic preload dependence of the continuous-flow pumps, which, unlike the native ventricle, lack a compensatory Frank-Starling reserve to buffer reductions in venous return. As a result, orthostatic intolerance in CF-LVAD patients can arise even in the setting of standard device parameters, minimal congestion, and normal Doppler-derived mean arterial pressures, making it challenging to diagnose and treat.

In healthy physiology, upright posture triggers a coordinated autonomic response involving baroreceptor activation, sympathetic vasoconstriction, and venous recoil to preserve cardiac filling and blood pressure. CF-LVAD support disrupts these mechanisms at multiple levels. First, continuous reduced pulsatile flow diminishes cyclic strain on carotid and aortic baroreceptors, leading to diminished afferent signaling and impaired sympathetic activation during postural stress.^3^, ^4^ The reduction in shear stress to the vascular wall—driven by reduced-pulsatile flow—promotes significant chronic vascular remodeling, characterized by aortic fibrosis, stiffening and structural changes, primarily due to increased fibrillar collagen deposition.^5^ Second, the loss of pulsatile shear stimuli reduces endothelial nitric oxide production and increases vascular stiffness, while promoting chronic activation of the sympathetic nervous system and renin-angiotensin-aldosterone axis, potentially worsening baroreflex-mediated vasoconstrictor responses.^6^ Third, gravitational blood pooling in the splanchnic and lower-extremity venous beds, physiologically addressed by baroreflex-mediated vasoconstriction, becomes more pronounced when adequate autonomic compensation is absent. Finally, CF-LVADs are highly preload-dependent: when venous return falls, the resulting increase in the upstream pressure gradient across the pump can cause low flows and symptomatic hypotension, which may occur independently of overt LVEDD reduction or suction events. See Figure 1.

**Figure 1:**
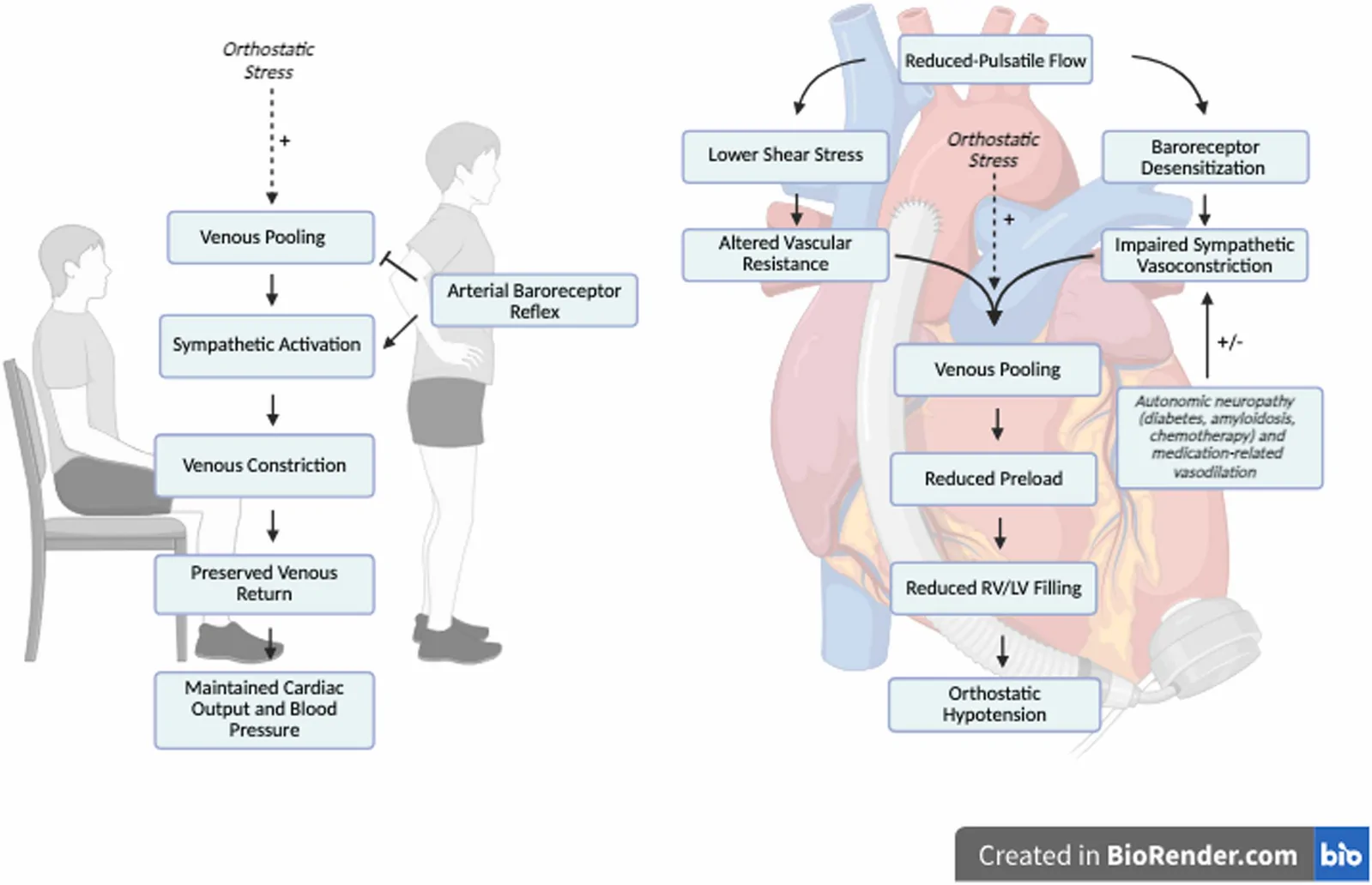
Mechanism of Normal Upright Physiology vs Autonomic Dysfunction-Predominant Failure.

Together, these factors make LVAD-associated OH a unique circulatory disorder situated at the intersection of autonomic dysfunction, altered vascular mechanics, and device-dependent preload physiology. Mechanistic clarity is essential as autonomic dysfunction-predominant failure can clinically resemble RV failure with positional hemodynamic decline, yet their treatments differ substantially. Posture-linked imaging and device readings provide a practical approach to differentiate these mechanisms at the bedside. This perspective piece integrates current physiology, LVAD hemodynamic evidence, and autonomic research to provide a comprehensive mechanistic framework for OH during CF-LVAD support.

Clinically, LVAD-associated orthostatic hypotension is not merely a nuisance finding. It is linked to falls, syncope, suction events, low-flow alarms, heart failure hospitalizations, and impaired quality of life. Since apparently similar presentations can arise from fundamentally different physiology, phenotype-agnostic treatment (for example, empiric volume loading in a patient with RV-limited forward flow) may exacerbate congestion or worsen symptoms. A practical, mechanism-based framework is therefore essential to help busy LVAD clinicians rapidly identify the dominant driver of orthostatic intolerance and match therapy to the underlying pathophysiology.

## Pathophysiological mechanisms of orthostatic intolerance during continuous-flow support

### Roles of baroreceptor dysfunction

Arterial baroreceptors rely on pulsatile stretch for afferent signaling. During CF-LVAD support, reduced pulse pressure and diminished systolic-diastolic oscillation lead to lower baroreceptor firing at rest, via under-stimulation in a dose-response relationship, and impaired responsiveness to postural change or limited reflex reserve.^3^ There is no specific threshold of pulsatility below which orthostatic hypotension invariably occurs (one study of CF-LVAD patients with pulse pressures as low as 14 ± 2 mmHg demonstrated preserved baroreflex sensitivity at −3.75 ± 0.79% mmHg compared to matched controls),^7^ rather any degree of reduced pulsatility contributes to baroreceptor dysfunction. This contrasts with classic neurogenic OH paradigms, where sympathetic activity is simply low or absent due to structural autonomic pathway degeneration, yielding a similar outcome. Furthermore, human microneurography studies show markedly elevated muscle sympathetic nerve activity (MSNA) at baseline in CF-LVAD patients. Direct microneurographic evidence indicates that the sympathetic reflex arc itself is not blunted during postural stress in CF-LVAD patients (MSNA rises appropriately, and in some cohorts is markedly augmented, with head-up tilt, and sympathetic baroreflex slope has been reported as comparable to matched controls^7^, ^8^). Rather, the functional impairment lies downstream: cardiac baroreflex sensitivity is reduced, and the fixed-output nature of the pump limits the ability to translate elevated sympathetic drive into an effective increase in stroke volume or blood pressure.^6^, ^9^ The chronically elevated resting MSNA in this population appears to reflect baroreceptor unloading from reduced pulse pressure, resetting sympathetic outflow to a higher set point, a process that is at least partially reversible with restoration of pulsatility.^7^, ^10^ Notably, most of this direct evidence derives from HeartMate II– and HVAD-era cohorts. Whether this pattern generalizes to HM3, with its intrinsic artificial pulse, has not been directly studied and remains an important question for future investigation.

### Chronotropic incompetence

Chronotropic incompetence (CI), the inability to appropriately increase heart rate in response to physiologic stress, is another prevalent manifestation of autonomic dysfunction in CF-LVAD recipients, observed in approximately 44% of patients and associated with reduced exercise capacity independent of beta-blocker therapy.^11^ Although CF-LVAD flow is relatively insensitive to heart rate at rest, an appropriate chronotropic response remains an important compensatory mechanism during postural transitions and ambulation, helping offset transient reductions in stroke volume. Patients with CI, whether due to intrinsic sinus node dysfunction, autonomic failure, or rate-limiting medications, may therefore be less able to compensate for the abrupt preload reductions accompanying upright posture, compounding orthostatic symptoms in the autonomic-predominant phenotype. Clinicians should consider CI as a contributing factor when evaluating recurrent or refractory orthostatic symptoms, particularly when reviewing rate-limiting medications as part of the broader medication rationalization discussed below.

### Venous capacitance and regional blood pooling

Total blood volume (TBV) is functionally divided into two pools: stressed blood volume (SBV), which generates vascular wall tension and drives venous return, and unstressed blood volume (UBV), which fills the vascular space without generating pressure. SBV normally constitutes approximately 30% of TBV. The distribution between these compartments is dynamically regulated by venous tone, primarily through sympathetic innervation of the splanchnic vasculature, which accounts for >40% of total body norepinephrine spillover.^12^ Advanced heart failure (HF) is characterized by a pathologic shift of blood from the unstressed to the stressed compartment, with total vascular capacity reduced by up to ~50% in chronic HF models. This means the splanchnic venous reservoir—which normally serves as a dynamic buffer during orthostatic stress—is already depleted even before LVAD implantation. The chronically elevated sympathetic tone to these splanchnic capacitance vessels leaves minimal reserve for further venoconstriction upon standing (where approximately 500–1000 mL of blood shifts into the splanchnic and lower-extremity venous reservoirs^13^), fundamentally predisposing the patient to preload insufficiency. In CF-LVAD patients, impaired sympathetic activation allows disproportionate venous pooling, causing reductions in right atrial (RA) pressure, LV filling, and, subsequently, device flow.^14^ This physiology contributes to the marked ventricular underfilling and increases the likelihood of suction events.

### Intrinsic preload sensitivity of CF-LVADs

CF-LVAD flow is governed by pump speed and the pressure differential across the pump—specifically, the gradient between the aortic pressure (afterload) and the left ventricular pressure (preload).^15^ During the transition from supine to upright posture, reductions in venous return lower the LV pressure. This widens the LV-to-aorta pressure gradient against which the pump must work, resulting in reduced estimated pump flow. Concurrently, this lead to a dramatic increase in this pressure gradient, as well as an abrupt decrease in LVEDD, smaller LV chamber size, and a higher risk of inflow cannula obstruction or suction events. Studies demonstrate that centrifugal flow output is minimally influenced by heart rate or autonomic tone but highly sensitive to variations in preload and passive venous return.^16^ In comparison to the healthy left ventricle, HeartMate III (HM3) CF-LVADs are more sensitive to reduced preload and less responsive to increased preload (reflecting impaired Frank-Starling mechanism).^17^ Thus, even modest postural reductions in venous return can produce symptomatic hypotension.^18^

### Endothelial and neurohumoral alterations

Reduced pulsatile flow (influenced by pump speed, native contractility and opening of the aortic valve) alters shear stress, endothelial nitric oxide synthesis, and vascular resistance.^19^ CF-LVAD patients demonstrate increased plasma norepinephrine and renin-angiotensin-aldosterone activation, suggesting chronic autonomic imbalance.^3^, ^6^ One study of 29 LVAD patients revealed heart rate and blood pressure variability, with attenuated nocturnal blood pressure dipping, using 24-hour monitoring, highlighting the chronic autonomic dysregulation and abnormal diurnal regulation in these patients.^20^ However, this study focused on patients with HeartMate II, since no ambulatory blood pressure monitors have been validated for HM3 that generate reduced-pulsatile flow. These maladaptations may amplify orthostatic intolerance and worsen responsiveness to physiologic stress.

### Right ventricular dysfunction

Right ventricular dysfunction itself is proposed as a driving mechanism for orthostatic hypotension in patients with CF-LVADs, influenced by key pump performance factors. Firstly, the LV and interventricular septum significantly contribute to RV contractility.^21^ CF-LVADs impair right ventricular contraction by shifting the septum leftward during acute LV unloading and by altering the normal contractile pattern of the myocardium due to its physical anchoring in the apex.^22^ Furthermore, LVAD implantation leads to RV dilation, spherical remodeling and increased wall stress over time, compounded by augmented cardiac output which increases venous return and RV preload, that ultimately reduces its mechanical efficiency.^23^ Finally, while LV unloading can improve RV contractility via reduced pulmonary artery (PA) pressure, residual or pre-existing pulmonary hypertension leads to RV-PA uncoupling and ultimately RV dysfunction.^23^

## Clinical presentation: Two dominant physiologic phenotypes of orthostatic intolerance

Although clinical presentations vary, two physiology-driven phenotypes can explain most cases of orthostatic intolerance in CF-LVAD patients: autonomic dysfunction-predominant failure and RV-failure/limited forward flow. Distinguishing these mechanisms is essential, as therapies beneficial in one phenotype may worsen the other. It is important to recognize that many CF-LVAD patients may have combined RV dysfunction and autonomic impairment, as both are common in this population, which can make these mechanisms challenging to distinguish in practice.

### Autonomic dysfunction-predominant failure

The following clinical vignette represents a typical presentation for an autonomic dysfunction-predominant phenotype: *An older patient with long-standing diabetes and a CF-LVAD presents with lightheadedness on standing despite normal resting hemodynamics. Tilt echocardiogram shows massive splanchnic pooling with a small, collapsible IVC, preserved RV size and function, and marked reductions in RA pressure and LV filling during upright posture*.

These patients experience exaggerated venous pooling, marked reductions in right atrial (RA) pressure and LV filling pressure upon standing, and a drop in mean arterial pressure without congestion, due to mechanisms described above.^9^ Autonomic testing often demonstrates autonomic failure, reduced blood pressure response to Valsalva (as evidenced by an absent or blunted phase IV blood pressure overshoot and absent reciprocal heart rate changes),^6^ and blunted sympathetic reflexes. Management centers on preload augmentation, compression therapy, and vasoconstrictors, further described below.^9^

### RV failure-limited forward flow

In contrast, the following clinical vignette demonstrates a typical presentation for a patient with right ventricular limited forward flow: *a patient class 3–4 heart failure and pulmonary hypertension on CF-LVAD support develops presyncope with ambulation. Upright echocardiography demonstrates a dilated, hypokinetic RV, rising RA pressure, reduced cardiac index, and a plethoric, non-collapsible IVC with little change in venous capacitance.*

As described, most patients with this phenotype exhibit positional increases in RA pressure, reduced cardiac index, RV dilation, and a plethoric, non-collapsible IVC during tilt or upright imaging. This is largely due to a mismatch between venous return and relative RV output, leading to a vicious cycle of reduced LV preload and ultimately reduced LVAD flow.^22^ In the setting of an already failing RV, gravitational stress further unmasks the RV’s inability to increase forward flow. Treatment focuses on RV-directed strategies: optimizing preload, reducing afterload, using pulmonary vasodilation in select cases, and adjusting device speed, further described below.^24^

### Overlap and mixed phenotypes

In practice, many CF-LVAD patients do not fall cleanly into a single category. Mixed phenotypes are common, particularly in those with autonomic impairment and limited RV reserve, where both exaggerated venous pooling and impaired RV forward flow contribute to orthostatic symptoms. A key contributor to this overall is the altered preload dependence of the failing RV itself. Whereas a normal RV operates on the ascending limb of the Frank-Starling curve with substantial functional reserve, tolerating wide fluctuations in venous return, a dysfunctional RV operates on a flattened, rightward-shifted curve. This has two consequences relevant to orthostatic physiology: the failing RV has diminished capacity to augment output when preload increases, and it is simultaneously more vulnerable to reductions in preload, having little functional reserve to draw upon. As a result, even the modest gravitational venous pooling characteristic of the autonomic-predominant phenotype can be poorly tolerated in a patient with limited RV reserve, producing a component of RV-limited forward flow superimposed on autonomic failure. Conversely, in patients with primary RV dysfunction, superimposed autonomic impairment can exacerbate positional instability by removing the compensatory venoconstriction that would otherwise help maintain RV filling within its narrow tolerable range. This bidirectional vulnerability underscores why mixed phenotypes are common in practice and reinforces the value of the two-phenotype framework as anchors for identifying the dominant contributor, even when RV preload sensitivity and autonomic dysfunction coexist.

## Diagnostic evaluation: Integrated approach to orthostatic intolerance in LVAD patients

A physiology-anchored diagnostic strategy begins with excluding reversible contributors to orthostatic symptoms, such as hypovolemia, infection, anemia, or medication-related vasodilation when taking a thorough history, before attributing symptoms to LVAD-specific physiology. Once these are addressed, the core of evaluation centers on determining how venous return and RV reserve respond to gravitational stress, and whether symptoms arise from autonomic-predominant preload failure or RV-predominant orthostatic intolerance**.** Diagnostic modalities for differentiating these two phenotypes are described below. See Figure 2.

**Figure 2:**
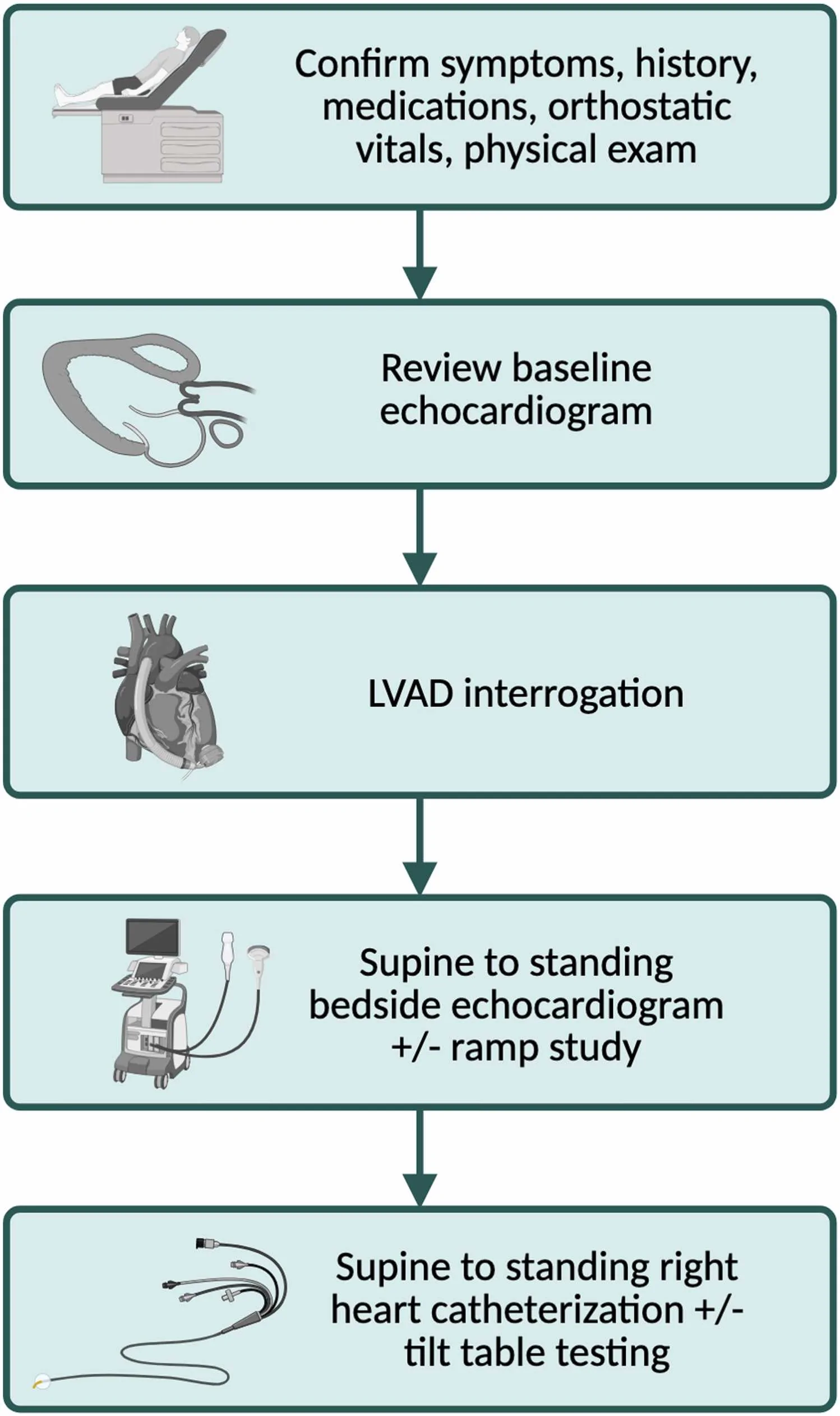
Stepwise Approach in the Diagnosis of Orthostatic Hypotension in LVAD Patients.

### Bedside assessment and orthostatic measurements

While orthostatic hypotension is classically defined as a fall in systolic blood pressure ≥20 mm Hg and/or diastolic blood pressure ≥10 mm Hg within 3 min of standing (or head-up tilt),^1^ applying these strict numerical thresholds to CF-LVADs is inherently flawed. In CF-LVAD recipients, reduced pulsatile flow complicates automated cuff and pulse-based methods; when a pulse is absent, Doppler opening pressure is often used as a practical surrogate for mean arterial pressure (MAP), with many guidelines targeting a MAP of roughly 70–90 mm Hg (often ≤80–85 mm Hg).^25^ However, relying solely on Doppler MAP changes fails to capture the unique hemodynamics of continuous-flow support and can easily lead clinicians to miss the underlying mechanism of a patient’s symptoms. Therefore, we propose decoupling the clinical syndrome of orthostatic intolerance from classic blood pressure thresholds. In the CF-LVAD population, the diagnosis is best conceptualized through a “symptom-plus-physiology” paradigm. Because CF-LVADs are exquisitely sensitive to venous return, positional pooling can precipitate rapid declines in LV filling, pump flow, and cerebral perfusion when Doppler MAP changes are modest, delayed, or technically unmeasurable due to poor flow signals. In this context, symptoms of cerebral hypoperfusion—rather than an arbitrary numerical drop—should drive the clinical evaluation.

Orthostatic vital signs, using Doppler ultrasound with sphygmomanometer, remain a reasonable starting point and should be performed after the patient has been supine for 5 min and then after standing, as this method has been shown to be more sensitive than sitting-to-standing for detecting OH.^26^ Tilt-testing can be utilized for this assessment especially when delayed OH is suspected, initial bedside evaluation is non-diagnostic, or if the patient is unable to safely perform active standing. There is likely significant benefit to extending testing beyond 3 min after standing or tilt if suspicion is high; of the 230 patients with OH in one retrospective study, only 46% exhibited OH within 3 min, 15% exhibited OH between 3 and 10 min and 39% exhibited OH after 10 min of tilt-testing.^27^

Ultimately, clinicians must recognize that the absence of a classic MAP drop does not exclude orthostatic hypotension in an LVAD recipient. Symptoms such as lightheadedness or presyncope, when coupled with physiological markers of positional failure (e.g., clinical cues of autonomic dysfunction, ^26^ echocardiographic reductions in LV size, or telemetry-derived drops in pump flow), are sufficient to establish the diagnosis and proceed with echocardiography and invasive testing to determine the specific phenotype.

### Echocardiography

Echocardiography provides rapid, non-invasive physiologic insight during postural stress and often mirrors the invasive hemodynamic signatures. Ideally, bedside echocardiography is performed while the patient is supine, sitting and standing, after waiting 3–5 min with each positional change. In the autonomic dysfunction-predominant phenotype, the IVC appears small at rest and becomes markedly collapsible with standing or sitting upright. This change is accompanied by a visible reduction in LV end-diastolic dimension, reflecting diminished venous return and pronounced preload limitation. Because CF-LVADs are inherently preload-sensitive, even modest decreases in venous return can result in meaningful reductions in pump inflow, predisposing patients to suction phenomena.

The RV failure phenotype exhibits a plethoric, poorly collapsing IVC that remains engorged even when upright, consistent with elevated RA pressures and impaired forward flow. While LVEDD may still decrease slightly due to gravitational influences, the RV typically appears dilated with limited contractile reserve and abnormal septal motion, reinforcing the notion that postural intolerance in these patients originates primarily from right-sided circulatory constraints. In LVAD patients whose resting IVC size may be chronically abnormal, the posture-linked change in IVC caliber and LV chamber size is often more informative than any static measurement alone.^2^, ^28^

### Invasive hemodynamics

Invasive hemodynamic assessment using right heart catheterization during postural stress such as with supine to standing positional changes or tilt table testing, reveals two reproducible physiologic phenotypes among LVAD recipients with orthostatic symptoms.^9^ In patients with autonomic-predominant preload failure, the transition from supine to upright posture typically results in a fall in right atrial and pulmonary capillary wedge pressures (PCWP) or maintenance of already low filling pressures, reflecting a failure of sympathetic vasoconstriction and excessive venous pooling. Throughout this manuscript, 'low' filling pressure is used in a relative, within-patient sense — reflecting a fall in, or failure of, RA pressure and PCWP to rise appropriately from the patient's own supine baseline — rather than a fixed population-wide numeric threshold, given substantial baseline variability across CF-LVAD patients. For reference, RA pressure <5 mmHg and PCWP <10 mmHg are commonly used as representative low-normal values in the broader hemodynamic literature, but the postural trend, rather than an absolute cutoff, is the feature we propose as diagnostically relevant. Cardiac index often remains stable or declines only modestly because forward flow is limited primarily by inadequate preload rather than by intrinsic cardiac dysfunction. This pattern mirrors known impairments in baroreflex sensitivity and sympathetic vasoconstrictor reserve seen in continuous-flow LVAD patients.^3^, ^6^

Patients with RV-predominant orthostatic intolerance demonstrate an rise in or persistently elevated RA pressure with standing or tilting, often disproportionate to any change in PCWP, reflected in an elevated RAP/PCWP ratio, accompanied by a marked decline in cardiac index.^14^, ^22^ This pattern reflects preserved venous return to the right heart with failure of the RV forward output, rather than left-sided congestion. This contrasts with late right heart failure driven by inadequate LV decompression (e.g. low pump speed, significant AR/MR, pump malposition, or residual pulmonary hypertension), in which PCWP can be elevated due to the chronic left-sided filling pressure elevation, and in which increasing pump speed, rather than reducing it, may be counterproductive if it worsens septal shift.^21^ The contrasting hemodynamic behaviors of these two phenotypes highlight the core conceptual point that identical bedside symptoms may arise from fundamentally opposite physiologic mechanisms.

### Device parameters

Device parameters provide a valuable surrogate window into physiologic changes during posture shifts, particularly in patients in whom cuff blood pressure measurements are unreliable or uninterpretable. There is growing interest in leveraging LVAD telemetry as a form of noninvasive, continuous physiology monitoring; for example, Ruoff and colleagues^29^ recently demonstrated that dynamic analysis of pump parameter responses can characterize posture- and activity-related hemodynamic transitions in CF-LVAD patients. In this context, the pulsatility index (PI) can be especially informative when interpreted alongside the broader clinical picture. While PI is traditionally viewed as a primary marker of residual native left ventricular contractility, its mathematical derivation as a quotient means it must not be interpreted in strict isolation. Because PI behavior can vary significantly based on device type, fixed speed setpoints, native LV contractility, severe hypertension (high afterload states), and arrhythmias, posture-related changes in PI, estimated flow, and power should be viewed as patterns rather than absolute rules. For instance, while hypovolemia typically lowers PI due to reduced LV filling, severe preload reduction can cause a paradoxical effect. During standing, a decline in LV preload that provokes septal shift and impending suction may cause the minimum pump flow to plummet; this artificially widens the flow amplitude, causing the PI to rise even if the mean arterial pressure is low.¹⁰ These specific PI spikes can be a signature of the autonomic phenotype, in which inadequate autonomic compensation leads to abrupt reductions in venous return. Conversely, in the RV-failure phenotype, PI may fall modestly or remain relatively unchanged with standing, yet suction alarms can still occur as septal shift and RV dilation impede LV inflow.

It is important to contextualize these telemetry findings within the evolution of LVAD technology. While foundational research on pump hemodynamics includes legacy devices (such as the HeartMate II and HeartWare HVAD), the current clinical landscape is dominated by the HM3. The HM3 features an intrinsic artificial pulse (the Lavare cycle) designed primarily to minimize pump stasis and hemocompatibility-related adverse events. While one might theoretically expect this intrinsic pulsatility to mitigate orthostatic hypotension, clinical experience demonstrates that severe autonomic dysfunction and orthostatic intolerance remain highly prevalent in the HM3 population.^9^ Furthermore, although the specific proprietary algorithms used to calculate PI vary among device generations, the underlying physiologic principle—that acute preload reduction and impending suction precipitate mathematical spikes in PI—remains clinically applicable across platforms.

Pump flow tracings also respond to posture: decreases in estimated flow with standing reflect reduced preload, whereas erratic flow patterns or power fluctuations may indicate more advanced ventricular interaction abnormalities. Correctly interpreted, LVAD telemetry functions as a physiologic amplifier**,** translating subtle posture-related hemodynamic shifts into immediately visible signals and highlighting the emerging role of remote, data-driven approaches in CF-LVAD management.^3^

Importantly, interpretation of device data must always be contextualized within the broader physiologic picture, as “over-speeding” the pump can mimic or exacerbate preload failure, while “under-speeding” may unmask RV dysfunction via elevated RV afterload and adverse septal positioning. LVAD speed optimization is therefore guided not by arbitrary targets but by a mechanistic understanding of preload sensitivity versus RV capacity, ensuring that device settings support rather than aggravate the dominant physiologic limitation.^8^, ^20^ Echocardiogram-guided ramp studies can be used as an adjunct to right heart catheterization when the mechanism or phenotype remains unclear and if speed optimization is being considered as part of a comprehensive management strategy. See Figure 3.

**Figure 3:**
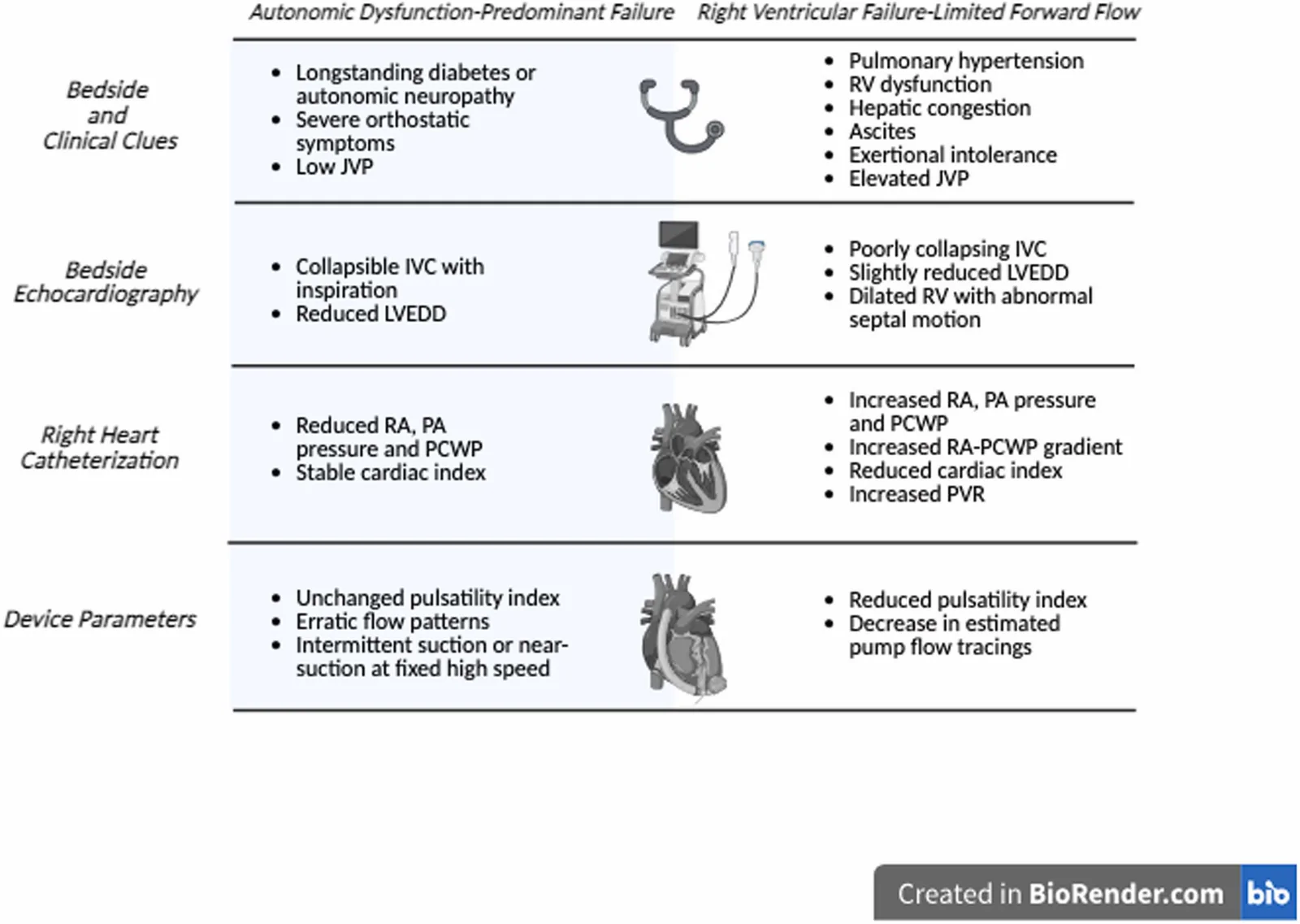
Diagnostic Differences in Autonomic Dysfunction-Predominant Failure vs Right Ventricular Failure-Limited Forward Flow.

Continuous ambulatory PA pressure monitoring (e.g., CardioMEMS) offers a complementary, longitudinal signal that may help distinguish these phenotypes outside the confines of a single clinic visit or formal postural study. Because PA diastolic pressure approximates PCWP, trends in ambulatory PA pressure with activity or diurnal variation may parallel the postural filling-pressure patterns observed on invasive hemodynamics: a fall in PA pressure with upright activity would be consistent with the autonomic-predominant phenotype and could serve as an early signal to prompt volume or postural countermeasures before symptoms or low-flow alarms occur, whereas persistently elevated or non-falling PA pressures with activity would be more consistent with RV-limited forward flow and might prompt afterload-reduction or RV-directed strategies rather than volume administration. Given its capacity for remote, longitudinal monitoring,^30^ CardioMEMS may be particularly well suited to capturing intermittent or activity-dependent physiology that episodic clinic-based testing could miss and could be integrated with PI and echocardiographic findings to refine phenotype assignment and guide therapy over time. This application remains conceptually proposed rather than validated, and prospective study of CardioMEMS-guided management of CF-LVAD-associated orthostatic intolerance is an important direction for future research.

## Management: Strategies based on physiology

### Autonomic-predominant preload failure

Management of autonomic dysfunction-driven orthostatic intolerance focuses on augmenting preload and supporting upright vasoconstrictor tone. Liberalizing oral fluid and salt intake, and using fludrocortisone cautiously when appropriate, help expand intravascular volume. Mechanical countermeasures such as compression stockings or abdominal binders reduce venous pooling in the splanchnic and lower-extremity compartments, regions that can sequester up to 40% of circulating volume during standing.^19^, ^21^ Engaging in physical maneuvers including leg-crossing, stooping, squatting and tensing the muscles of the legs, arms, buttocks or whole body have been shown to increase blood pressure in patients with orthostatic intolerance,^31^ but this has not been specifically studied in patients with LVADs. Head-up tilt sleeping may also be considered as an adjunct to reduce nocturnal natriuresis and improve morning orthostatic tolerance, although supporting data derive primarily from non-LVAD OH populations and should be extrapolated cautiously.^32^ Behavioral strategies including slow postural transitions, avoidance of prolonged standing or heat exposure, and small frequent meals to limit postprandial pooling can further reduce symptom burden.

Pharmacologic vasoconstrictors, such as midodrine, provide targeted adrenergic support to counteract gravitational venous pooling. Equally important is avoiding “over-speeding” the LVAD, which can intensify the pump’s preload dependence and precipitate suction or low-flow states during upright stress. In this phenotype, “optimal” hemodynamics may involve tolerating slightly higher filling pressures to preserve venous return and LV filling rather than pursuing aggressively low RA or wedge pressures.

Background medications should be reviewed carefully. In patients with severe OH, non-essential vasodilators including diuretics, high-dose angiotensin converting enzyme inhibitors, angiotensin II receptor blockers, angiotensin receptor-neprilysin inhibitors, nitrates, and sodium-glucose cotransporter-2 inhibitors should be reduced or temporarily held if they exacerbate hypotension or intravascular depletion. These decisions require nuance in LVAD patients, where GDMT also supports long-term outcomes; however, phenotype-aware prioritization of upright perfusion is critical in autonomic-predominant preload failure. The overarching therapeutic principle is to stabilize venous return and reduce reliance on a failing autonomic system to maintain upright perfusion.

A particular challenge arises in patients with coexisting resting or supine hypertension, in whom withdrawal of antihypertensive therapy to treat OH risks inducing resting hypertension, which is itself a concern in CF-LVAD patients given its association with increased afterload, aortic insufficiency progression, pump thrombosis risk, and stroke.^33^ In these patients, strategies include taking antihypertensives in the evening or at bedtime to blunt supine hypertension while preserving daytime blood pressure needed for upright perfusion as well as prioritizing non-pharmacologic countermeasures including compression garments, abdominal binders, and slow position changes. Ultimately, the treatment choice should be individualized, taking into account the risks of resting hypertension and the severity of orthostatic symptoms.

### RV failure-limited forward flow

Management of RV-limited orthostatic intolerance aims to optimize RV preload without provoking congestion, reduce RV afterload, and adjust LVAD speed to preserve favorable interventricular interactions. Gentle volume optimization can be helpful, but overdistension exacerbates septal shift and worsens LV filling. Pulmonary vasodilators, when used judiciously, reduce RV afterload in patients with reactive pulmonary hypertension or exercise-induced pulmonary pressure elevations and have been associated with improved outcomes.^22^

LVAD speed must be titrated thoughtfully: excessive speed shifts the septum leftward, impeding RV function and worsening the cycle of impaired preload delivery. In this phenotype, reducing speed may improve interventricular dependence and restore more balanced filling.^10^ Thus, “optimal” hemodynamics differ from the autonomic phenotype—prioritizing RV unloading and septal position over strict preload augmentation.

As in autonomic-predominant cases, medication regimens should be rationalized. GDMT may be beneficial when it reduces pulmonary congestion and RV afterload,^34^ but beta-blockers and vasodilators should be used cautiously if they precipitate bradycardia, hypotension, or worsen RV contractile reserve. Diuretics should be titrated to relieve congestion without collapsing preload. Non-pharmacologic measures such as compression and behavioral modifications may still help symptoms but should not distract from the central need to restore RV forward flow. Finally, digoxin can be used for positive inotropy as a last-resort adjunct, given the mixed evidence for this medication in this setting.^35^

Contributing factors including arrhythmias, hypoxia, pulmonary hypertension, and residual volume overload should be addressed promptly to preserve RV reserve. Ultimately, successful management depends on aligning device settings and medical therapy with the dominant physiologic limitation rather than pursuing generic hemodynamic targets (Figure 4).

**Figure 4:**
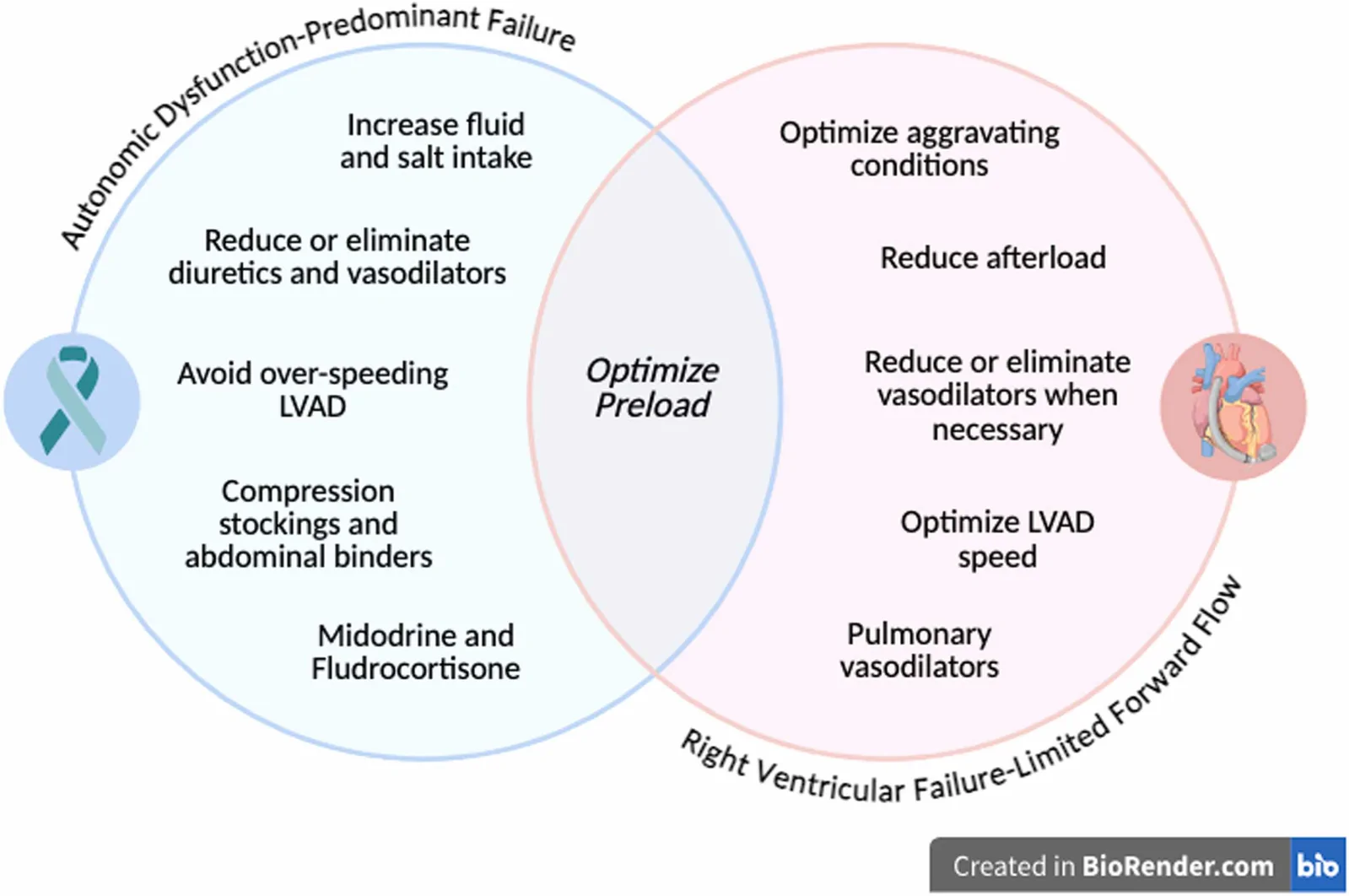
Physiology-based Management for Autonomic-Predominant Preload Failure vs Right Ventricular Failure-Limited Forward Flow.

### Future directions and research gaps

Despite growing recognition of OH in CF-LVAD patients, prospective studies defining prevalence, mechanisms, and phenotype-specific treatment strategies are lacking. Formal evaluation of autonomic testing, posture-linked imaging, and LVAD hemodynamics in integrated diagnostic pathways are needed. Neuromodulation strategies, notably baroreflex activation therapy (BAT; e.g., Barostim), that has shown durable improvements in symptoms and functional capacity in selected HF patients via increased parasympathetic tone, may hold promise for selected autonomic-predominant patients but require dedicated study in the LVAD population given baseline baroreflex unloading from reduced pulsatility.^36^, ^37^ Finally, opportunities may exist for remote physiologic monitoring and AI-driven pump speed adaptation to dynamically respond to posture and activity, potentially reducing orthostatic events and improving quality of life; however, more research is needed to elucidate the safety and regulatory oversight of this.

## Conclusion

Orthostatic intolerance during CF-LVAD support arises from a complex interaction between diminished pulsatility, baroreflex impairment, venous capacitance changes, and intrinsic device preload sensitivity. A physiology-first understanding, centered on baroreflex biology, venous return mechanics, and RV-LV interaction, allows for accurate diagnosis and targeted therapy. Recognizing the two dominant mechanistic phenotypes enhances clinical decision-making and may reduce morbidity associated with low-flow alarms, suction events, and functional impairment. Future work should validate standardized physiologic testing and develop intervention trials targeting autonomic dysfunction in CF-LVAD patients.

## Financial Disclosure Statement

This research did not receive any specific grant from funding agencies in the public, commercial, or not-for-profit sectors. The authors report no relevant disclosures or conflicts of interest regarding the publication of this manuscript.

## Declaration of Generative AI and AI-assisted technologies in the writing process

During the preparation of this work the authors used Google AI Studio in order to proofread and fix errors in the manuscript. After using this tool/service, the authors reviewed and edited the content as needed and take full responsibility for the content of the publication.

## Declaration of Competing Interest

The authors declare that they have no known competing financial interests or personal relationships that could have appeared to influence the work reported in this paper.
